# Congenital duodenojejunal junction obstruction; report of two rare cases

**DOI:** 10.1016/j.ijscr.2024.109766

**Published:** 2024-05-16

**Authors:** Jayalaxmi Shripati Aihole

**Affiliations:** Indira Gandhi Institute of Child Health, Bangalore, Karnataka. India

**Keywords:** Duodenojejunal junction obstruction, Duodenal web, Malrotation, Gastric outlet obstruction, Duodenal atresia

## Abstract

**Introduction and importance:**

Congenital obstruction of duodenojejunal junction is a rare unexplored pathologic entity. Most of the cases reported so far are regarding extrinsic band or narrower attachment of ligament of Treitz, which will be presenting with vomiting in neonates and children without malrotation.

**Case presentation:**

Author is reporting here two rare cases of congenital intrinsic duodenojejunal junctional obstruction and their management in toddlers.

**Clinical discussion:**

Duodenojejunal junction, is an embryologically, pathologically and radiographically, yet unexplored region except anatomically and surgically. Only few pathologies have been described in this region so far

**Conclusion:**

An abnormal embryogenesis could be speculated and contemplated to be the reason for such rare congenital duodeno jejunal junctional obstruction which should be managed promptly as per required surgical techniques.

## Highlights

• Congenital duodenal obstruction due to duodenal web (type I) commonly reported in the second part of duodenum, rarely reported in other parts of duodenum as well as at rest of the bowel.
• Gray and Skandalakis have classified congenital duodenal obstructions into 3 types- I, II, and III. The type I or membranous duodenal obstruction (mucosal diaphragm, or web) has been quoted to account for Iin 2500 to 10.000 births.
• congenital duodenojejunal junction web has been rarely reported so far, which can be managed by simple surgery called duodenojejunostomy without requiring tapering and without any morbidity.
• Occasional vomiting and atypical radiographic signs due to fenestrated web leading to atony and ineffective peristalsis of the duodenum can contribute to delayed presentations and hence the morbidity.

## 1 Introduction

Congenital duodenal obstruction most commonly occurs due to malrotation, atresia, stenosis and annular pancreas, whereas, congenital obstruction of the distal duodenum at or near the ligament of Treitz are due to high fixation or hyper fixation bands [1,2]. Congenital intrinsic duodenojejunal junction obstruction is rarely reported along with its pathology. The resultant dilated atonic hypertrophic long segment of duodenum is surgically challenging to manage requiring either subtotal resection or tapering [1,2].

## 2 Case reports

### 2.1 Case 1

**Fig. 1:**
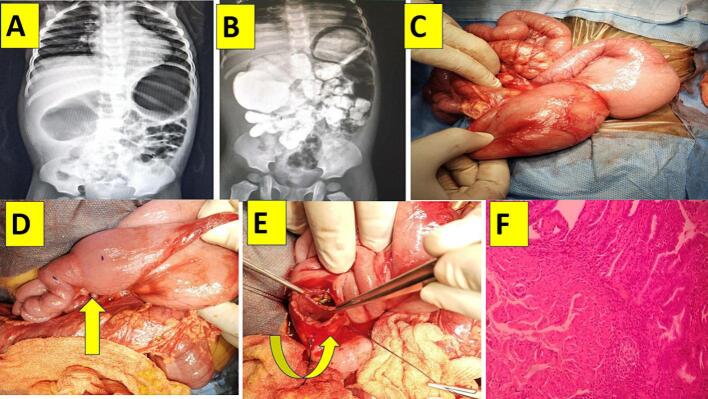
A - Plain abdominal radiography showing dilated stomach and duodenum. B - Contrast study showing dilated stomach and entire duodenum with delayed emptying. C - Intra operative image showing dilated stomach and megaduodenum. D - Upward arrow-pointing at, DJ junction web; mega duodenum and distal normal jejunum. F - Low power 20X×-histopathologic view showing mucosa, submucosa and few fibres of muscularis mucosae.

Two year three months old male child coming from western part of India, was born by full term cesarean section to non-consanguineously married couple after twin gestation, with a birth weight of 2.75 kg; asymptomatic and thrived well till two years of age, following which child used to have occasional bilious vomiting twice in a week since 3months. On admission, child was clinically and hemodynamically stable with normal laboratory parameters with 9kg weight. Upper gastrointestinal contrast study done, showed hugely dilated stomach and duodenum with delayed emptying (Fig. 1A, B). There was no significant family history neither baby had any associated congenital anomalies. On exploration there was, hugely dilated stomach and whole of the duodenum (first, second, third and fourth part) associated with collapsed jejunal loops and rest of bowel, without any evidence of malrotation. After kocherisation, entire duodenum was inspected and palpated. There was a sharp cut off precisely at duodenojejunal flexure, after releasing of ligament of Treitz with mucosal web was felt and seen with luminal disparity (Fig. 1C, D). Longitudinal enterotomy was made over an anti-mesenteric border across the duodenojejunal junction crossing the web. Circumferential mucosal web having tiny pin point orifice was exposed and excised anterolaterally, leaving behind the posterior wall with cautery and sent for biopsy. Longitudinal enterotomy was closed transversely in a single layer with absorbable vicryl suture (Fig. 1E). Feeds were started after 48 hours and child was discharged on day 5 uneventfully. Child is doing well after a year of follow up.

### 2.2 Case 2

**Fig. 2:**
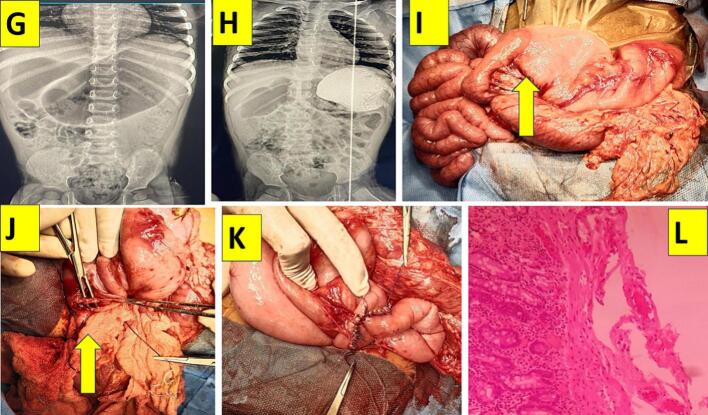
G - Plain radiography showing dilated stomach with duodenal shadow. H - Upper gastrointestinal contrast study showing dilated stomach with diluted dye in the dilated duodenum. I - Dilated duodenum (forth part) and collapsed jejunal loops. Yellow arrow pointing at web area, a sharp demarcation. J - Duodenojejunotomy - revealing circumferential web with pin point orifice admitting only tip of the artery forceps. G - Duodenojejunostomy in single layer. H - Low power 20-histopathologic view showing mucosa, submucosa and few fibres of muscularis mucosae with inflammatory cell infiltrate.

One-year ten months old male child from southern part of India, was born by normal vaginal full-term delivery with a birth weight of 2.5kg was having on and off bilious vomiting since birth. Child was 9 kg with normal hematological parameters and clinically stable. Baby did not any significant family history or any congenital anomalies. Contrast study revealed hugely dilated stomach and duodenum with delayed emptying. On exploration and after kocherisation, hugely dilated stomach and entire duodenum was seen and after release of ligament of Treitz, a mucosal web was felt and seen exactly at duodenojejunal junction flexure (Fig. 2G, H, I, J). The longitudinal enterotomy of around 1.2 cm across the web and partial excision of the web and transversely closed in a single layer with absorbable suture vicryl suture. Post operative period was uneventful, feeds were started after 48 h and baby was discharged on day 5 (Fig. 2K, L).

Both babies are doing well. Histopathology revealed duodenal mucosa with hypertrophied muscularis mucosae without muscularis propria (Figs. 1F, 2L).

Work has been reported in line with the SCARE criteria.

## 3 Discussion

Embryologically, duodenum develops from caudal part of foregut bud and cephalic part of midgut during fourth to sixth week of intrauterine life. Initially its lumen is obliterated by the rapid growth of its own epithelial cells, subsequently to get recanalized by vacuolization at around twelfth week; failure of this process, leads to intrinsic duodenal obstruction like atresia. Hence cephalic portion of primary intestinal loop develops into third and fourth part of duodenum and Jejunum [1,2]. This differentiates congenital duodenal atresia from atresia in the rest of the intestines, which result from an intrauterine vascular accident. Though some authors now believe that vascular events may be the underlying cause of duodenal atresia as well as Jejunal atresia [1,2].

This abnormal embryological process leaves behind a web, made of only the mucosa and the submucosa layers, the muscularis layer being absent. A duodenal web is a thin, circumferential, web-like structure, may be complete or less frequently having small tiny orifice, seen most commonly in the second part of duodenum rarely reported in other parts [3,4].

This abnormal embryological process could be speculated and contemplated to be the reason for duodeno jejunal junction obstruction in these two cases.

Louw et al, suggested such cases in which the duodenum shows evidence of dilation and hypertrophy with no evidence of malrotation, labeled as Congenital megaduodenum (duodenal diameter of 5 cm or more) may require imbrications or a tapering duodenoplasty to avoid prolonged duodenal ileus [[4], [5], [6]].

Occasional vomiting and atypical radiographic signs due to fenestrated web leading to atony and ineffective peristalsis of the duodenum can contribute to delayed presentations and hence the morbidity [5,6].

Though congenital duodenal obstruction due to mucosal diaphragm, atresia and stenosis have been well reported in literature, however only few cases of congenital duodenojejunal junction obstruction have been reported so far, either due to abnormal attachment of ligament Treitz or rarely due to intrinsic webs; hence requiring tapering duodenoplasty and duodnojejunostoplasty, rarely duodenojejunostomy [1,2,4].

Author had two male children with duodenojejunal junctional intrinsic mucosal web, presenting with occasional bilious vomiting since birth. Surprisingly both children had grown well with normal hematological parameters. On exploration, hugely dilated stomach and entire duodenum were noticed (Figs. 1C, D, 2G, H, I). The anterolateral excision of web and duodenojejunostomy in single layer was done without requiring tapering duodenoplasty uneventfully. Both children tolerated the procedure well and are doing well on follow up.

## 4 Conclusion

Duodenojejunal junction, is an embryologically, pathologically and radiographically yet unexplored region except anatomically and surgically. Only few pathologies have been described in this region so far.

Author is reporting here two such rare cases, managed effectively by simple surgical technique.

Hence, sharing experience of such rare cases, can help to broaden the knowledge of the attending clinicians to effectively diagnose and manage such cases without morbidity and mortality.

1. Patient consent: Consent to publish the case report has been obtained from parents. This report does not contain any personal information that could lead to the identification of the patient.”
2. Funding-None
3. Authorship: Include the following statement: All authors attest that they meet the current ICMJE criteria for Authorship.—yes
4. Work has been reported in line with the SCARE criteria

## Consent

Both verbal and written consent for publication has been taken from parents a copy of this written consent is available for review by editor in chief of this journal on request.

## Ethical approval

Ethical approval has been taken from Indira Gandhi Child Health Institute Bengaluru Karnataka India on 5.1.24.

## Funding

None.

## Author contribution

Dr Jayalaxmi Shripati Aihole has contributed to the article.

## Guarantor

Dr Jayalaxmi Shripati Aihole.

## Research registration number

None.

## Conflict of interest statement

None.
